# Dual-sided EEG electrode signals have varying correlations that depend on movement characteristics

**DOI:** 10.1038/s41598-025-31782-9

**Published:** 2025-12-13

**Authors:** Jinfeng Li, Helen J. Huang

**Affiliations:** 1 Guangdong Provincial Key Laboratory of Development and Education for Special Needs Children, Lingnan Normal University, Zhanjiang, 524048 Guangdong China; 2 Department of Mechanical and Aerospace Engineering, University of Central Florida, Orlando, FL 32816 USA; 3 Disability, Aging and Technology (DAT) Cluster, University of Central Florida, Orlando, FL 32816 USA; 4 Bionic Materials, Implants and Interfaces (Biionix™) Cluster, University of Central Florida, Orlando, FL 32816 USA

**Keywords:** Electroencephalography - EEG, Biomedical engineering

## Abstract

Mobile Electroencephalography (EEG) measures human brain activity during locomotion, extending brain dynamics research into real-world scenarios. However, EEG is highly susceptible to artifacts, and more reliable approaches are needed to attenuate motion artifacts in mobile EEG recordings. Dual-layer EEG, which records scalp EEG simultaneously with isolated motion artifact signals, presents a novel and promising approach. This method assumes that noise-biased EEG data captures the common noise as the isolated motion artifact data. The purpose of this study was to investigate the relationship between signals from both sides of the electrode and their relationship to movement. We developed a benchtop test platform where the top and bottom sides of a dual-sided electrode interfaced with conductive fabric. Using a robotic arm, we moved the dual-sided electrode with different directions, magnitudes, frequencies, and randomness. We quantified correlations between signals from the top and bottom sides to understand the relationship of the dual-sided signals. We also quantified correlations between movement and signals from both the top and bottom sides to examine the extent to which the signals are related to movement*.* Movement direction affected the correlation signs, and all correlation metrics scaled with movement magnitude rather than frequency. Increased movement randomness reduced top and bottom signal correlation. These findings contribute new insights into signal correlations of the dual-sided EEG electrode and could inform the development of improved dual-layer EEG algorithms and hardware innovations for real-world applications.

## Introduction

Motion artifacts that are caused by multiple contributors greatly compromise EEG signals during walking. EEG is easily contaminated by undesired noise that originates from electrophysiological or non-electrophysiological sources^1,2^. Electrophysiological artifacts arise from eye movement, heartbeat, and muscle activity, while non-electrophysiological sources include powerline noise, electromagnetic interference, and movement-related artifacts^3,4^. In particular, the motion artifacts have greater amplitude than electrocortical signal and can noticeably undermine EEG signal quality during mobile EEG studies such as walking due to the inevitable body and device movements^5,6^. The relative movement between recording electrode and scalp is the major contributor to motion artifacts. This movement leads to variations in the electrical coupling of skin-electrode interface and results in signal distortion^7,8^. More specifically, compared to horizontal movements, vertical movements along the axis perpendicular to the electrode surface cause larger fluctuations in contact impedance due to dynamic changes in contact pressure and area^7,9^, thereby contributing to stronger motion artifacts^10^. Cable sway is another main source of motion artifacts in wired EEG system^11,12^. Cable sway generates friction and deformation on the cable isolator, resulting in triboelectric noise^13,14^. In general, motion artifacts are direction dependent and increase with higher cyclical movement frequencies^12,15^ or faster walking speeds^10,16,17^, and substantially degrade EEG signal quality. The effective removal of motion artifacts is of great importance as it facilitates the interpretation of EEG signal.

Researchers have developed various hardware and software techniques to reduce motion artifacts. Continuous advances in EEG electrodes improve the quality of EEG recordings. Gel-based silver/silver chloride (Ag/AgCl) electrodes are the current gold standard for EEG recordings owing to their low skin-electrode impedance, high reliability, and good signal-to-noise ratio^18–20^. Thus, wet electrodes are more resistant to motion artifacts compared to dry electrodes. Active electrodes reduce cable-induced motion artifacts by amplifying the signal at the recording site with embedded amplifiers^20,21^. Integrating additional skin–electrode impedance^22,23^ or relative displacement^24^ monitor circuitry into the electrodes, increasing electrodes surface area^12^, or simply applying proper pressure to the electrodes^25^ can further improve EEG signal quality during motion. Novel electrode designs like tripolar concentric ring electrodes^26^, semi-dry electrodes^27^, and tattoo-like or textile-based flexible electrodes^28,29^ improve signal-to-noise ratio to varying degrees.

Apart from hardware approaches, software algorithms play a vital role in removing motion artifacts from EEG signals. Basic high-pass filters with cut-off frequencies of 0.5–2 Hz, depending on the movement speeds involved in studies, are usually used to attenuate motion artifacts before other artifact removal techniques^30,31^. Regression and adaptive filters can reduce motion artifacts in EEG signals by modifying the filter coefficients based on artifacts characteristics if one or more motion related reference channels are available^32–35^. When a reference signal is not available, Bayesian filters such as Kalman filters are feasible choices as they use probabilistic approach to estimate the EEG state based on recorded EEG data^36,37^. Artifact template regression and template correlation rejection procedures have also been used to remove gait-related motion artifacts from EEG during walking without a reference channel^38,39^. The most popular and widely acknowledged method for motion artifact removal is blind source separation, which reconstructs statistically independent sources from mixed signals without additional reference channel or prior information about the origin of signals^40^. Independent component analysis (ICA) is the most commonly used blind source separation method. ICA first linearly decomposes EEG recordings containing artifacts into independent components, then reconstructs the cleaned EEG data after removing the artifact components^41,42^. If a clean EEG baseline trial is available, artifact subspace reconstruction (ASR) uses principal component analysis decomposition and removes large-variance artifact components based on a predetermined threshold^43–45^. Mobile EEG studies often use combined algorithms to attenuate motion artifacts in EEG recordings during locomotion^46–54^, as none of the above-mentioned algorithms alone can be an optimal choice for motion artifact removal. However, special attention should be directed to the algorithms because neuronal signals may also be heavily attenuated with aggressive processing. Contrarily, signals that are not effectively cleaned may lead to misinterpretation of electrocortical activity. Studies that used a swim cap to block scalp EEG, thereby recording isolated motion artifacts, which were then processed using the same methods as those for scalp EEG, found independent components in brain areas that had spectral fluctuations coupled with gait events^10,55^.

Incorporating additional sensor measurements into EEG processing pipeline is a promising approach for mobile EEG artifact removal. Simultaneous recordings of accelerometer^56–58^, gyroscope^59^, or inertial measurement unit^60^, have been used along with ICA or machine learning to identify and remove motion artifacts induced by head movement^61^. However, the limited number of additional sensors did not individually correlate with EEG channels, as head movement differentially affects each EEG channel depending on the channel location on the scalp^62^. To tackle this problem, researchers have developed a more advanced technique, dual-sided EEG electrodes, that record scalp EEG simultaneously with isolated motion artifacts^63^. The motion artifact removal ability of the dual-layer EEG system was evaluated by moving an electrical phantom head that projects known simulated source signals^63,64^. The dual-layer EEG has also been used to study cortical dynamics during walking over obstacles^65^, walking at different speeds^66^, and playing table tennis^67,68^. The complex signal processing pipeline employed was based on the assumption that the dual-layer electrodes share common motion artifacts. However, the relationship between the signals recorded from the two sides of a dual-sided electrode in response to controlled movements of the surfaces that interface with the electrodes was not characterized. Determining this relationship may lead to a simpler and more effective artifact removal pipeline, and full utilization of the EEG data.

The purpose of this study was to evaluate dual-sided EEG electrodes and determine if the signals recorded from the two sides are highly coupled when moving with different combinations of directions, magnitudes, frequencies, and randomness. We hypothesize that signals recorded from the two electrodes of a dual-sided electrode would: 1) have higher correlations during vertical movements compared to horizontal movements, 2) have higher correlations during movements with larger magnitudes, 3) have higher correlations during movements with higher frequencies and have dominant frequencies equal to the movement frequencies of the electrodes, and 4) have lower correlations with more randomized movements.

## Methods

### Setup and protocol

**Fig. 1:**
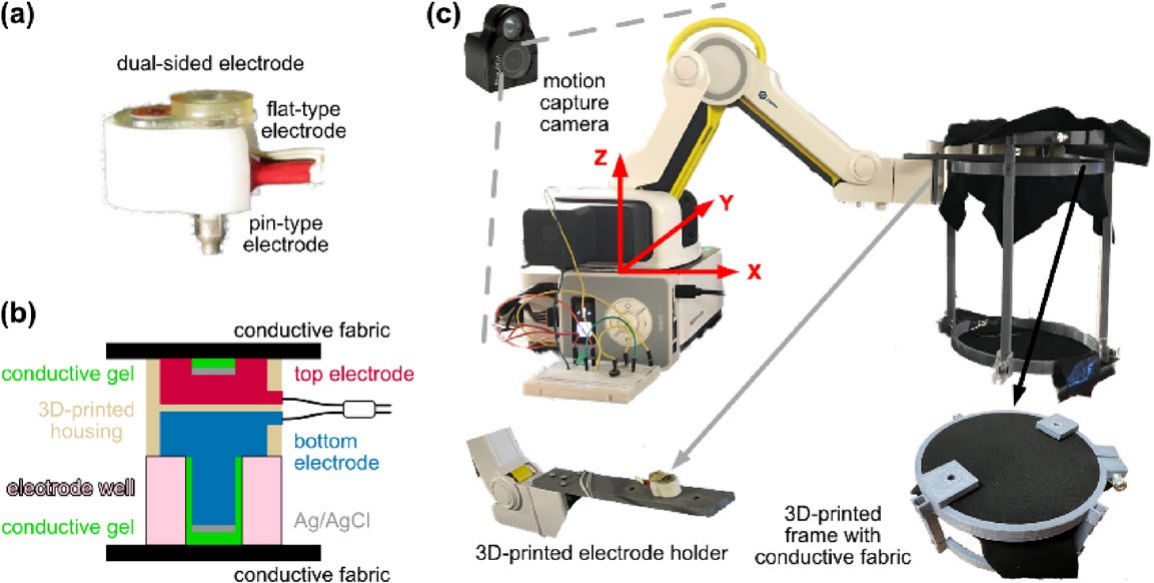
Setup and electrodes. (**a**) A dual-sided Electroencephalography (EEG) electrode in a 3D-printed housing. (**b**) Schematic of the dual-sided EEG electrode and its interfaces. (**c**) Robotic arm drove a 3D-printed electrode holder with a dual-sided EEG electrode interfaced with conductive fabric on both sides, while motion capture cameras recorded the movements.

We customized the dual-sided electrode (Fig. 1a) using BioSemi ActiveTwo hardware (ActiveTwo, BioSemi B.V., Amsterdam, the Netherlands). We used pin-type Ag/AgCl active electrode as the bottom electrode and inverted flat-type Ag/AgCl active electrode as the top electrode (Fig. 1a, b). The bottom electrode was mechanically coupled to, yet electrically isolated from, the top electrode via a 3D-printed housing, with wires from both electrodes bundled using tape (Fig. 1a, b). We attached one dual-sided electrode to a 3D-printed electrode holder (Fig. 1c). Each side of the dual-sided electrode was interfaced with a conductive fabric (EeonTex, LTT-PI-100, Marktek Inc., Chesterfield, MO, USA), which was attached to a 3D-printed frame (Fig. 1b, c). The conductive fabric served as an electrically conductive surface to mimic the skin-electrode interface in real-world EEG recordings. We did not introduce simulated EEG signals into the system, and the conductive fabric was not used to amplify electromagnetic interference. Motion artifacts were generated through the relative movement between the electrode and the conductive fabric surface.

We programmed a desktop robotic arm (DOBOT Magician Lite, Shenzhen Yuejiang Technology, Shenzhen, China) to systematically drive the electrode holder with different combinations of frequencies (1, 1.25, 1.5, and 1.75 Hz)^12,15,63^, directions (X: forward and backward, Y: left and right, Z: up and down), and magnitudes (1, 2, 3, and 5 mm). We also have random trials where the robotic arm moved randomly within a predetermined 3D space (dominant direction allowed movement range was always up to 5 mm, while non-dominant directions movement ranges were limited up to 0, 1, 2, 3, or 5 mm). The random trials lasted for 60 s, and the other trials contained 60 full movement cycles. We recorded the trials by the order of frequencies and ranges (from small to large)^15^.

We connected the dual-sided electrodes to two daisy-chained AD-boxes (ActiveTwo, BioSemi B.V., Amsterdam, the Netherlands) such that the signals from both sides of a dual-sided electrode were synchronized and recorded at 512 Hz. We attached four passive reflective markers (4 mm diameter) on the electrode holder and recorded the marker movements using a motion capture system (OptiTrack, NaturalPoint Inc., Corvallis, OR, USA, 13 Prime13W and 9 Prime13 cameras) with a sampling frequency of 240 Hz^69^.

### Data analysis

We performed data analysis in MATLAB (version 9.7, R2019b, MathWorks Inc., Natick, MA, USA).

#### Motion capture data

We low-pass filtered the marker trajectories using a fourth-order Butterworth filter with 12 Hz cut-off frequency^70^. We then extracted movement onset and ending events and divided the motion capture and electrode data into movement cycles for each trial. We used the middle 30 s of the random trials, and the middle 30 movement cycles of the cyclic trials for subsequent analyses. We normalized the amplitude of electrode movement for each trial using min–max normalization, scaling the values to the range of [−1, 1].

#### EEG electrode data

We bandpass filtered EEG electrode data from 0.5 to 59 Hz^12^, and normalized the amplitude of the electrode data for each trial to the range [−1, 1] using min–max normalization. We calculated the Pearson correlation coefficients: 1) between top and bottom electrode signals, 2) between electrode movement and top electrode signal, and 3) between electrode movement and bottom electrode signal. To mitigate the effect of sampling distribution skew when averaging correlation coefficients, we transformed the Pearson correlation values by applying Fisher’s Z transformation^71,72^. We interpreted the strength of Fisher’s Z transformed correlations in three levels using low (−0.49 to 0.49), moderate (0.5 to 0.99 or −0.99 to −0.5), and high (≥ 1 or ≤ −1)^73^.

We calculated the power spectral density (PSD) for each trial by using “spectopo” function with a 4000 ms Hamming window and 50% overlap^15^. We then calculated magnitude-squared coherence between top and bottom electrode signals using “mscohere” function. We also performed time–frequency analysis on each trial using “newtimef” function for both top and bottom electrode signals, with the average power across time within cycles of the stationary baseline trial as the spectral baseline.

#### Statistics

We performed statistics using SPSS (version 28) and JMP Pro (version 16) with the significance level of 0.05. We first excluded outliers beyond 3 standard deviations from the mean for each data set. We then used a Shapiro–Wilk W test, Levene’s test, and Mauchly’s test to check for the distribution normality, homogeneity of variance, and sphericity, respectively.

For the first three hypotheses evaluating the effects of movement direction, magnitude, and frequency, we performed three-way repeated measures analysis of variance (rANOVA) on each Fisher’s Z transformed correlation metric, with direction, magnitude, and frequency as factors. If a significant three-way interaction effect was found, we further examined the simple two-way interaction effects for each pair of factors (direction * magnitude, direction * frequency, magnitude * frequency) by the third factor, and if a significant simple two-way interaction effect was found, we further investigated the second-order simple main effects. If no significant three-way interaction effect was present, we proceeded to examine the two-way interaction effects for each combination of two factors (direction * magnitude, direction * frequency, magnitude * frequency). When a significant two-way interaction effect was detected, we checked the simple main effects. Finally, if significant second-order simple main effects or significant simple main effects were found, we then performed post hoc pairwise comparisons using Tukey’s Honest Significant Difference (HSD) tests, which account for multiple comparisons. We also performed linear regression with the “fitlm” function to investigate the relationship between magnitude and each Fisher’s Z transformed correlation metric.

For the fourth hypothesis regarding randomness, we performed one-way rANOVA on Fisher’s Z transformed correlation between top and bottom electrode signals with randomness as a factor by movement direction. We followed by post hoc pairwise comparisons using Tukey’s HSD if randomness was a main effect.

## Results

### Representative data

**Fig. 2:**
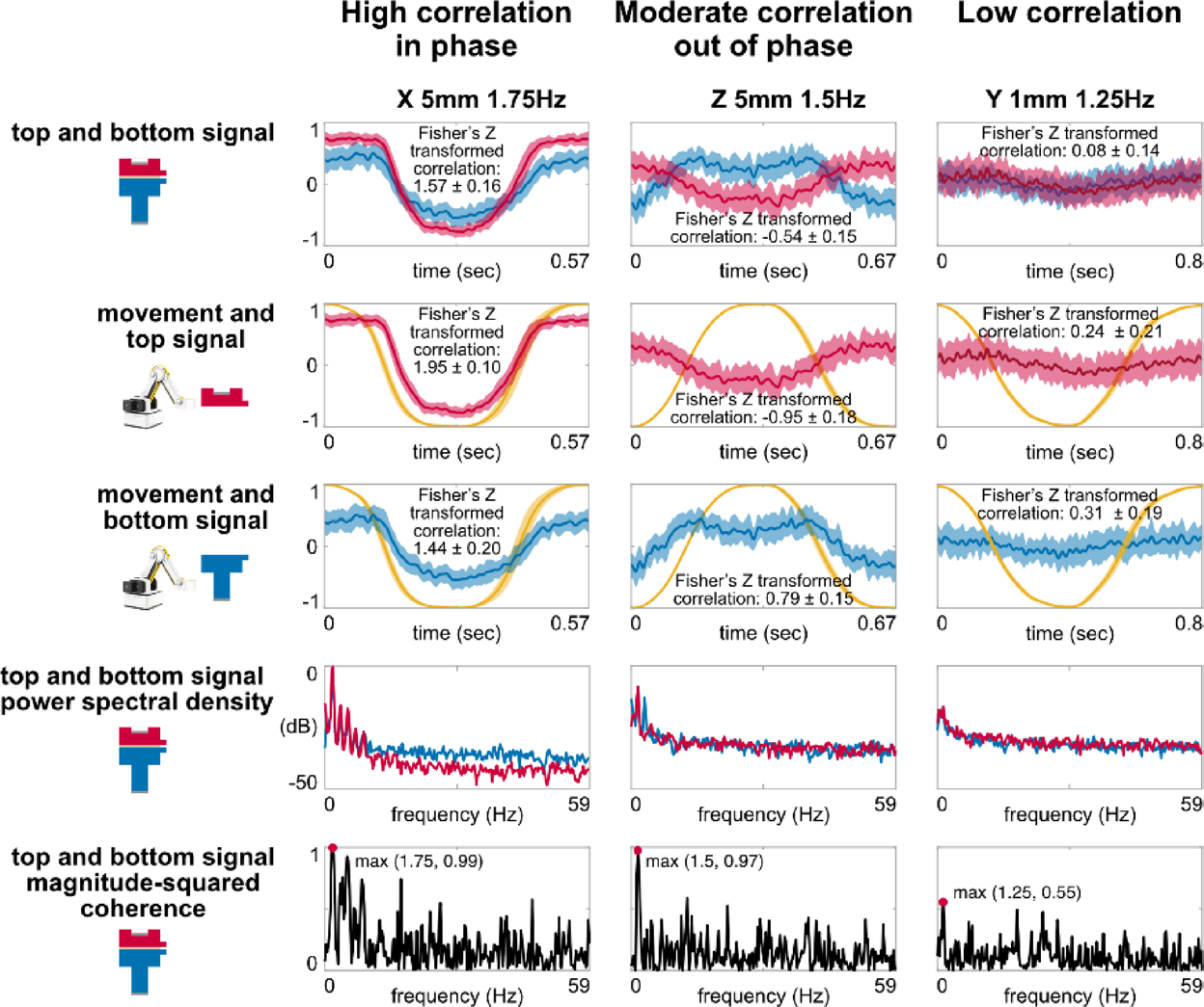
The dual-sided EEG electrodes showed three representative cases among the movements. High correlation in phase case showed high and positive top and bottom signal correlation, movement and top signal correlation, and movement and bottom signal correlation. High correlation out of phase case showed high and negative top and bottom signal correlation and movement and top signal correlation. Low correlation case showed low top and bottom signal correlation, movement and top signal correlation, and movement and bottom signal correlation. All three cases had peak top and bottom signal magnitude-squared coherence occurred at the corresponding movement frequency.

The dual-sided EEG electrode showed three representative cases among the movements (Fig. 2). The first case was high correlation in phase, such as X 5 mm 1.75 Hz movement, during which the trajectories of top and bottom electrode signals were in phase, produced high top and bottom signal correlation (1.57 ± 0.16), high movement and top signal correlation (1.95 ± 0.10), and high movement and bottom signal correlation (1.44 ± 0.20). The second case was moderate correlation out of phase, like Z 5 mm 1.5 Hz movement, where the top and bottom signals had opposite trajectories and induced moderate top and bottom signal correlation (−0.54 ± 0.15), moderate movement and top signal correlation (−0.95 ± 0.18), and moderate movement and bottom signal correlation (0.79 ± 0.15). Y 1 mm 1.25 Hz movement represented the third case of low correlation, where the top and bottom signals had no evident trends, resulting in low top and bottom signal correlation (0.08 ± 0.14), low movement and top signal correlation (0.24 ± 0.21), and low movement and bottom signal correlation (0.31 ± 0.19). Despite distinct features in the time domain, the peak top and bottom signal magnitude-squared coherence was located at the frequency corresponding to the movement frequency for each case.

### Direction effects

**Fig. 3:**
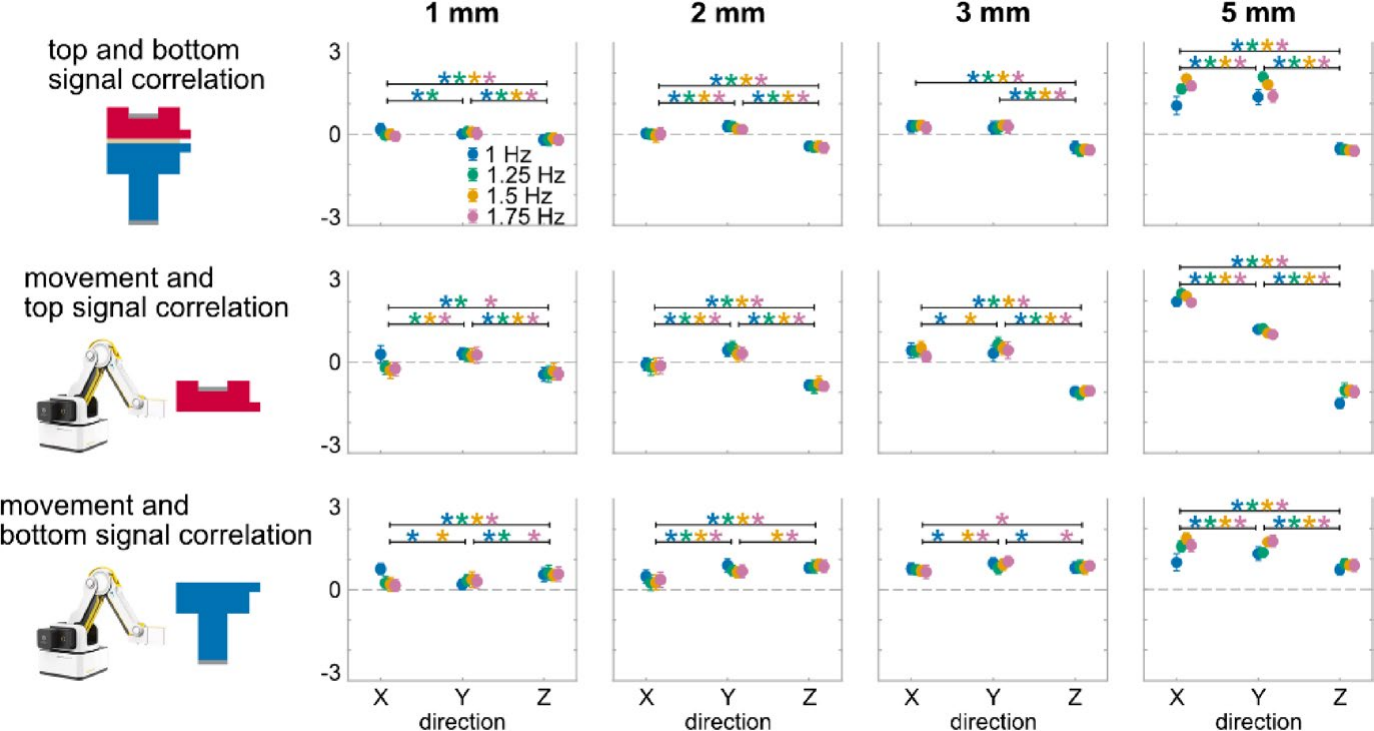
Averaged (mean and standard deviation indicated by dot and error bar) top and bottom signal correlation, movement and top signal correlation, and movement and bottom signal correlation of movements with 1 mm, 2 mm, 3 mm, 5 mm magnitude (four columns), in X, Y, Z direction (x axis), and 1 Hz (blue), 1.25 Hz (green), 1.5 Hz (yellow), 1.75 Hz (red) movement frequency. Vertical direction movements induced negative top and bottom signal correlation, and movement and top signal correlation. *: *p* < 0.05 and color-coded for the specific frequencies with significant differences between movement directions indicated by brackets.

Vertical direction movements induced negative top and bottom signal correlation, and movement and top signal correlation (Fig. 3). Direction, magnitude, and frequency had a significant three-way interaction effect on all metrics (top and bottom signal correlation: F (18, 486) = 17.86, *p* < 0.001; movement and top signal correlation: F (18, 486) = 7.75, *p* < 0.001; movement and bottom signal correlation: F (18, 486) = 14.88, *p* < 0.001). Direction and magnitude also had a significant simple two-way interaction effect on each metric for all frequency levels (F’s (4.2–6, 122.9–174) ≥ 43.80, *p*’s < 0.001), whereas direction and frequency had a significant simple two-way interaction effect on each metrics for all magnitude levels (F’s (3.8–6, 109–174) ≥ 3.33, *p*’s < 0.005) except for the top and bottom signal correlation during 2 mm and 3 mm movements (F’s (6, 168–174) ≤ 1.58, *p*’s > 0.14). Further, direction had a significant second-order simple main effect on each metric for all magnitude and frequency levels (F’s (1.4–2, 39.4–58) ≥ 7.20, *p*’s < 0.003) except for movement and bottom signal correlation during 3 mm 1.25 Hz movement (F (2, 58) = 2.13, *p* = 0.13). Almost all correlation metrics were positive when the movements were in horizontal directions (X and Y) regardless of movement magnitude and frequency. For movements in the vertical direction (Z), top and bottom signal correlation, and movement and top signal correlation were negative and significantly different from the corresponding values in X and Y directions (*p*’s < 0.039), whereas movement and bottom signal correlations remained positive.

### Magnitude effects

**Fig. 4:**
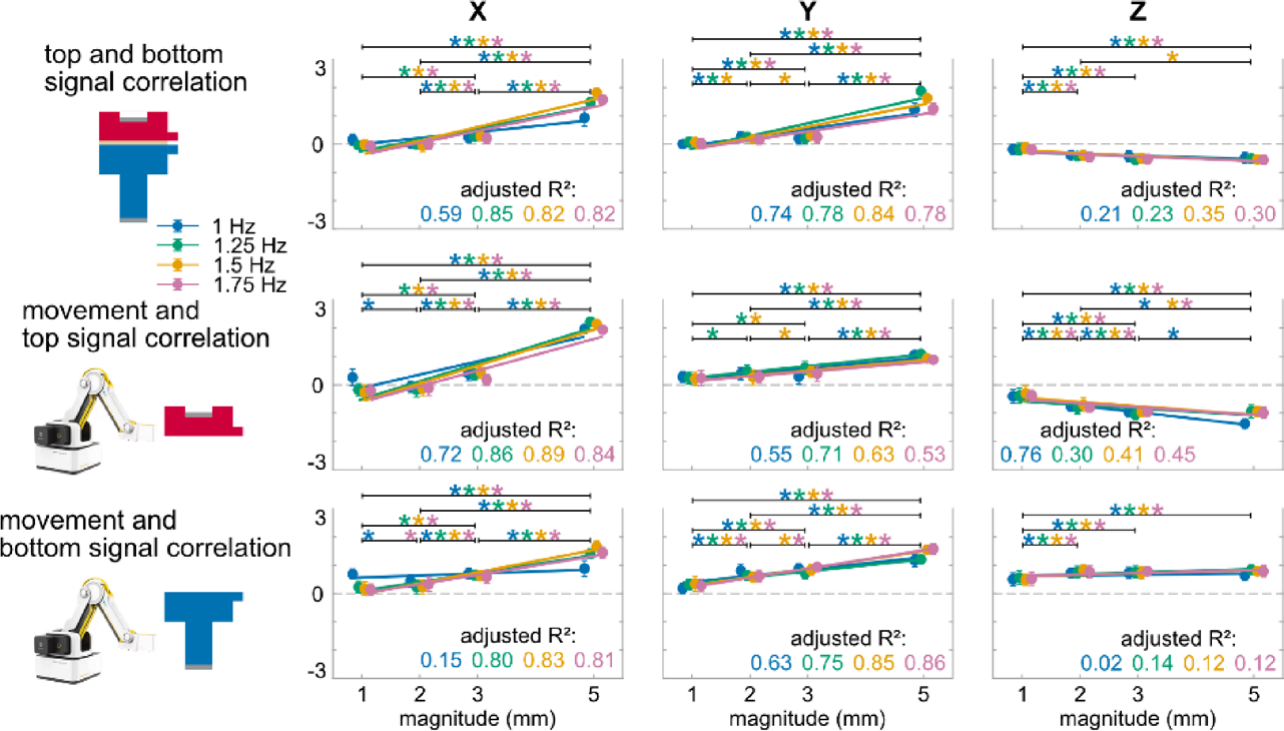
Averaged (mean and standard deviation indicated by dot and error bar) top and bottom signal correlation, movement and top signal correlation, and movement and bottom signal correlation of movements in X, Y, Z direction (three columns), with 1 mm, 2 mm, 3 mm, 5 mm magnitude (x axis), and 1 Hz (blue), 1.25 Hz (green), 1.5 Hz (yellow), 1.75 Hz (red) movement frequency. All correlation metrics scaled linearly with movement magnitude. The solid line indicates the linear regression fit. *: *p* < 0.05 and color-coded for the specific frequencies with significant differences between movement magnitudes indicated by brackets.

All correlation metrics scaled with movement magnitude (Fig. 4). Following the significant three-way interaction effect, magnitude and direction had a significant simple two-way interaction effect on each metric for all frequency levels (F’s (4.2–6, 122.9–174) ≥ 43.80, *p*’s < 0.001), while magnitude and frequency had a significant simple two-way interaction effect on each metric for all direction levels (F’s (5.3–9, 149–261) ≥ 3.65, *p*’s < 0.001) except for the top and bottom signal correlation and the movement and bottom signal correlations during movements in Z direction (F’s (9, 252–261) ≤ 1.60, *p*’s > 0.11). Magnitude had a significant second-order simple main effect on each metric for all direction and frequency levels (F’s (2.1–3, 58.2–87) ≥ 9.20, *p*’s < 0.0001). All correlation metrics generally deviated more from zero as movement magnitude increased, with almost all correlation metrics during 3 mm and 5 mm movements being significantly different from those during 1 mm movements (*p*’s < 0.028). Linear regression analysis revealed that all correlation metrics exhibited significant linear relationships with movement magnitude (adjusted R^2^ range: 0.12–0.89, *p*’s < 0.0001), except for the movement and bottom signal correlation during 1 Hz movement in Z direction (adjusted R^2^ = 0.02, *p* = 0.08).

### Frequency effects

**Fig. 5:**
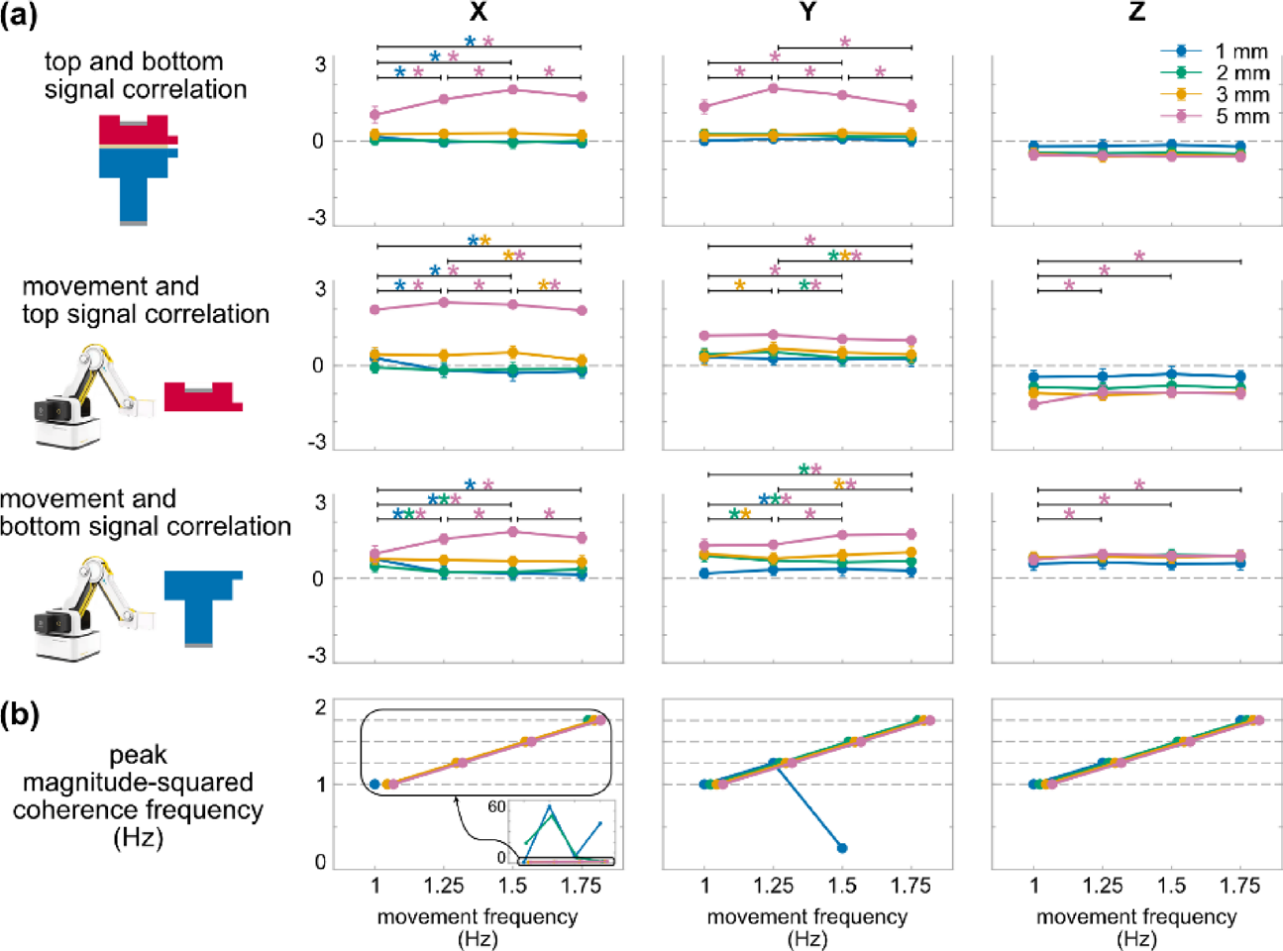
(**a**) Averaged (mean and standard deviation indicated by dot and error bar) top and bottom signal correlation, movement and top signal correlation, and movement and bottom signal correlation of movements in X, Y, Z direction (three columns), with 1 Hz, 1.25 Hz, 1.5 Hz, 1.75 Hz movement frequency (x axis), and 1 mm (blue), 2 mm (green), 3 mm (yellow), 5 mm (red) movement magnitude. Movement frequency had no evident effect pattern on any correlation metric. *: *p* < 0.05 and color-coded for the specific magnitudes with significant differences between movement frequencies indicated by brackets. (**b**) The frequency of peak magnitude-squared coherence (indicated by dot) for each movement condition.

Movement frequency had no evident effect pattern on any correlation metric (Fig. 5a). Despite the significant direction * magnitude * frequency interaction effect, simple frequency * magnitude, and simple frequency * direction interaction effects on all correlation metrics, frequency only had a significant second-order simple main effect on top and bottom signal correlation during X 1, 5 mm, and Y 2, 5 mm movements (F’s (2.6–3, 74.5–87) ≥ 2.86, *p*’s < 0.043), movement and top signal correlation during X 1, 3, 5 mm, Y 2, 3, 5 mm, and Z 5 mm movements (F’s (2.1–3, 57.8–87) ≥ 5.12, *p*’s < 0.004), movement and bottom signal correlation during X 1, 2, 5 mm, Y 1, 2, 3, 5 mm, and Z 5 mm movements (F’s (2.5–3, 73.6–87) ≥ 3.09, *p*’s < 0.032). No clear patterns were observed, despite some significant differences of correlations between movements at different frequencies.

Top and bottom signals had dominant frequencies equal to the movement frequencies (Fig. 5b). The peak magnitude-squared coherence frequencies for top and bottom signals matched the movement frequencies in 81.25% of the conditions (39 out of 48, exceptions were X 1 mm 1.25 Hz, X 1 mm 1.5 Hz, X 1 mm 1.75 Hz, X 2 mm 1 Hz, X 2 mm 1.25 Hz, X 2 mm 1.5 Hz, Y 1 mm 1.5 Hz, Y 1 mm 1.75 Hz, and Z 1 mm 1.5 Hz movements).

### Randomness effects

**Fig. 6:**
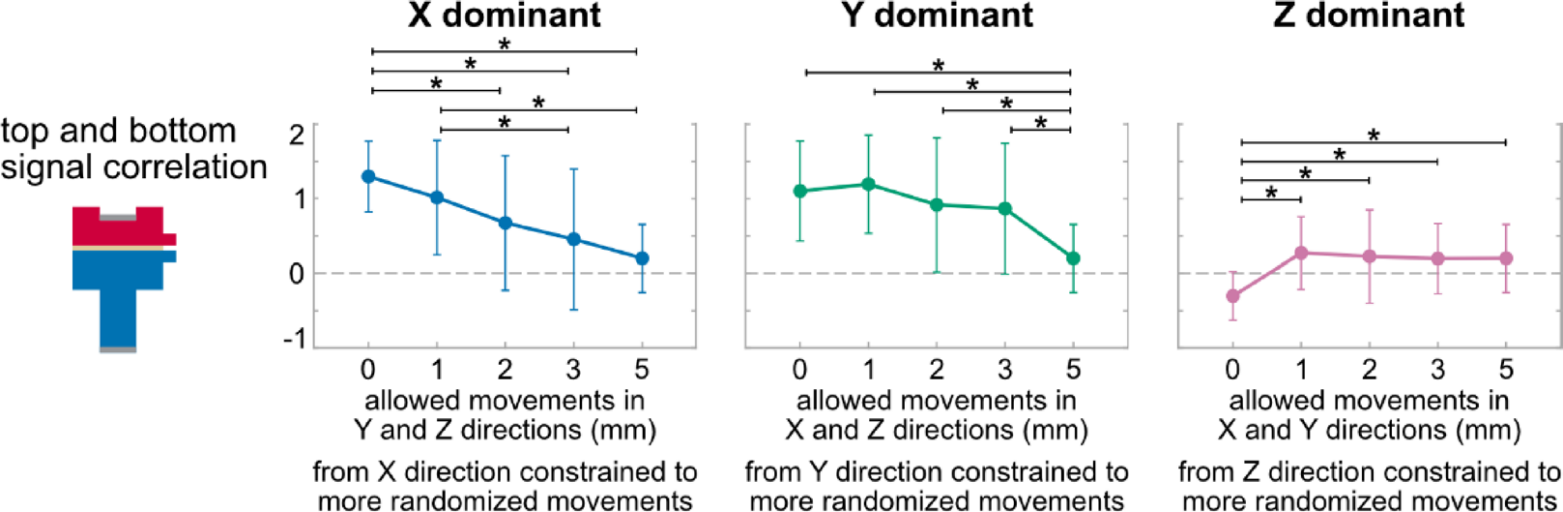
Averaged (mean and standard deviation indicated by dot and error bar) top and bottom signal correlation of movements in X, Y, Z dominant direction (three columns), from constrained to more random movement conditions (horizontal axis: 0, 1, 2, 3, 5 indicates allowed movement range in millimeters for the non-dominant directions, e.g., X dominant indicates allowed movement in X direction is always up to 5 mm, while allowed movements in Y and Z directions are limited up to 0, 1, 2, 3, or 5 mm). More randomized movements decreased top and bottom signal correlation. *: *p* < 0.05 and with significant differences between movement conditions indicated by brackets.

More randomized movements decreased top and bottom signal correlation (Fig. 6). Randomness had a significant main effect on top and bottom signal correlation in all directions (F’s (3.5–4, 101.3–116) ≥ 7.32, *p*’s < 0.0001). Top and bottom signal correlations were positive when movements were constrained in the X and Y dominant directions and were significantly different compared to the reduced correlations of movements with increased randomness (*p*’s < 0.038). Top and bottom signal correlations were negative when movements were constrained in the Z dominant direction and were significantly different compared to the relatively stable correlations of movements with more randomness (*p*’s < 0.002).

### Time–frequency analysis

**Fig. 7:**
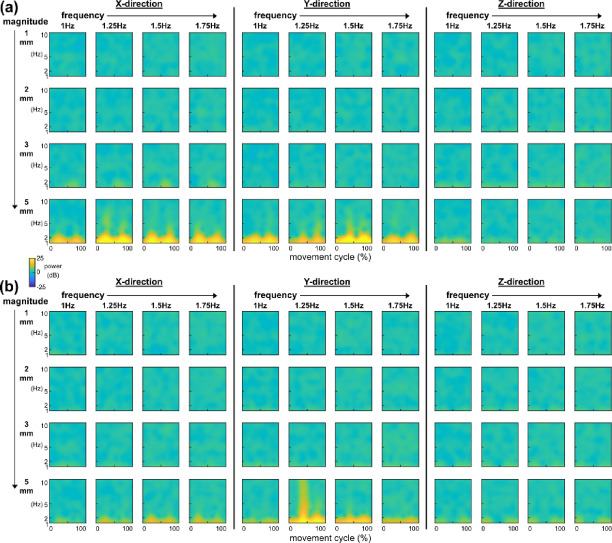
(**a**) Averaged time–frequency plots for top electrode signals of movements in X (columns 1–4), Y (columns 5–8), Z (columns 9–12) direction, with 1 Hz, 1.25 Hz, 1.5 Hz, 1.75 Hz movement frequency, and 1 mm (row 1), 2 mm (row 2), 3 mm (row 3), 5 mm (row 4) movement magnitude. (**b**) Averaged time–frequency plots for bottom electrode signals under the same movement conditions. Yellow indicates increased spectral power, and blue indicates decreased spectral power relative to stationary baseline. Large magnitude movements induced increases in low-frequency power.

Time–frequency analysis showed that large magnitude movements induced increases in low-frequency power (Fig. 7a, b). For both top and bottom electrode signals, power bursts below 2 Hz emerged during 3 mm movements and became more pronounced during 5 mm movements across all directions and frequencies. Low-frequency power began to increase with the onset of movement, and most peak power occurred around 25% and 75% of the movement cycle, corresponding to the instants of maximum movement velocity.

## Discussion

We investigated how movement direction, magnitude, frequency, and randomness affect the signals recorded from two sides of a dual-sided EEG electrode. Top and bottom electrode signal correlation, as well as movement and top signal correlation, were positive during horizontal movements but negative in vertical movements. All correlation metrics scaled with movement magnitude. Despite the lack of a clear pattern of frequency effects in time domain, the dominant frequencies of top and bottom signals were consistent with movement frequencies. Additionally, top and bottom signal correlation degraded when movements were less constrained. These results provide fundamental knowledge about the relationship between the signals recorded from the two sides of a dual-sided electrode in response to controlled movements, which could help guide the development of hardware and algorithmic solutions for attenuating motion artifacts or predicting motion characteristics from motion artifacts.

The first main finding is that vertical movements had a greater influence than horizontal movements on the sign of correlation between top and bottom electrode signals, and between movement and top signal. This may arise from the change of relative distance between the recording electrode and conductive fabric, which results in substantial impedance change^8,74–76^. This relative distance increased or decreased alternately during vertical movements, leading to out of phase dual-sided electrode signals and negative correlations. In contrast, the top electrode-fabric distance and the bottom electrode-fabric distance remained consistent during horizontal movements, introducing positive correlations. Vertical movements were exclusively employed in previous EEG motion artifact removal studies^15,63^ due to their dominant contributions to motion artifacts during human walking^10^.

Another main finding is that movement magnitude had an apparent linear scaling influence on all correlation metrics. We used the magnitudes of 1, 2, 3, and 5 mm and observed the expected scaling of correlations, which supported the second hypothesis that predicted higher correlations with larger movement magnitudes. This finding is consistent with a previous study that showed greater displacements reduced EEG signal-to-noise ratio^15^, and also in line with observations that faster walking speeds induce greater movements, consequently leading to more motion artifacts^10,16,17^.

Interestingly, movement frequency barely had substantial influence on any correlation metric and did not support our third hypothesis, which predicted that higher movement frequencies would generate higher correlations. We proposed this hypothesis because greater movement frequencies induced more motion artifacts and decreased EEG signal quality^63^. Similarly, greater cable motion frequencies reduced EEG signal-to-noise ratio^12^, although we assumed diminished effects of cable sway in our study since our movements were applied to the electrode itself and barely involved substantial cable movements. However, another study using multiple movement magnitudes and frequencies showed that the degradation of EEG signal quality was only evident when the displacement increased up to 6 cm^15^, suggesting that a large enough magnitude may be needed to produce apparent frequency effects.

As expected, the peak magnitude-squared coherence between top and bottom electrode signals matched the movement frequency. This result is consistent with previous findings that the top side of dual-sided EEG electrode captured the platform movement frequency^63^, and mildly supports the spectral noise cancellation approach for cleaning EEG motion artifacts.

Unsurprisingly, adding randomness degraded the correlation between top and bottom electrode signals. This may be explained by the more disordered relative movements of the electrode with the fabric. Despite the randomness of the movement, constraining the movement in the horizontal directions induced positive top and bottom signal correlations, while restricting the movement in the vertical direction induced negative correlations, thus reiterating the influence of movement direction. Interestingly, when there were dominant movements in the vertical direction, increasing randomness in the horizontal directions did not further reduce the top and bottom signal correlation, indicating that vertical movement had a greater influence on the correlation compared to horizontal movements. This finding supports prior studies that showed the substantial contribution of vertical movements to motion artifacts^10,15–17^.

One limitation of this study is the asymmetric design of the dual-sided electrode. The top side was a flat electrode, while the bottom side was a pin electrode, resulting in different amounts of gel on the two sides. The more symmetric design of dual-sided electrode used in recent study^77^ was custom-made by the manufacturer and is not widely available. Using the same design as the study that proposed the dual-sided EEG approach was a reasonable starting point. Another limitation is that the conductive fabric was not the best material to mimic the electrical properties of human skin. Ballistics gelatine or agarose has shown better properties^78,79^, but it may not be convenient to incorporate these materials into human mobile EEG study. Our robotic arm had acceleration limits, which restricted the movement frequencies we could use for larger movement magnitudes.

Despite the aforementioned limitations, our findings provide insights into the practical applications of dual-sided EEG in real-world EEG recording during movement. The directional dependence of dual-sided EEG signal correlations suggests that adaptive denoising algorithms could incorporate direction-specific processing strategies, such as phase inversion, particularly for vertical movements. The linear relationship between movement magnitude and correlation metrics indicates that dynamic artifact thresholds could be implemented proportionally to detected displacement levels, potentially improving artifact rejection accuracy during varying movement intensities encountered in daily activities. While leveraging the differential effects of movement direction and magnitude on signal correlation may require sophisticated motion segmentation and classification, our finding that top and bottom signals consistently share dominant frequencies matching movement frequencies supports the development of more generalizable frequency-domain denoising algorithms. This further supports the spectral noise cancellation approach^63^, suggesting that dual-sided electrodes could attenuate motion artifacts even when movement characteristics vary unpredictably, as in real-world scenarios. Beyond artifact attenuation, the direction and magnitude specific signal features identified in this study may also be leveraged to predict movement characteristics directly from motion-contaminated EEG signals.

Based on these algorithmic insights, brain-computer interface (BCI) systems—where motion artifacts significantly compromise decoding accuracy—could implement adaptive and frequency-domain artifact removal strategies to enhance robustness in mobile or sport-related applications^80^, such as robotic exoskeleton control. Additionally, leveraging artifact-based features to predict movement characteristics may further enhance BCI performance by providing complementary motion-related signals. For wearable EEG systems, our results highlight the importance of electrode stabilization, especially along the vertical axis or radially on the head, where correlation patterns are most distinctive. This could be achieved through design enhancements such as more symmetric electrode configurations, reinforced mounts, compliant materials, or spring-loaded mechanisms to buffer vertical impacts^5,81^. Ultimately, these advancements—such as optimized denoising algorithms, artifact-based motion prediction techniques, and dual- sided electrode designs—may enable more robust and interpretable EEG monitoring in both daily and clinical settings, such as dynamic walking, sports training, and neurological rehabilitation, where motion artifacts have historically impeded reliable signal acquisition^67,82^.

Future work should validate these strategies in phantom models and human trials under various movement protocols^83^. By translating our benchtop findings to optimize both hardware designs and algorithms for motion artifact removal or motion characteristics prediction, dual-sided EEG techniques could bridge the gap between controlled laboratory recordings and dynamic real-world applications, thereby expanding the utility of mobile EEG for both research and clinical applications.

## Conclusions

We evaluated how different directions, magnitudes, frequencies, and randomness of movements affect dual-sided EEG electrode signals. Movement magnitude, rather than frequency, had scaling influence on all correlation metrics, but the dominant frequencies of top and bottom electrode signals matched with movement frequencies. Movement direction affected the sign of all correlation metrics except for correlation between movement and bottom signal. Greater randomness reduced top and bottom signal correlation. Our findings provide better understanding of dual-sided EEG electrode signal characteristics, which could inform the development of denoising algorithms, artifact-based prediction of motion characteristics, and optimized dual-sided electrode designs for improved EEG performance in real-world applications.

## Data Availability

The datasets generated during and/or analysed during the current study are available from the corresponding author on reasonable request.
